# Effectiveness of Transcranial Stimulation on Cognitive Abilities of Older Adults with Mild Cognitive Impairment

**DOI:** 10.3390/jcm14072472

**Published:** 2025-04-04

**Authors:** Juan Miguel Muñoz-Perete, Javier Cano-Sánchez, Yolanda Castellote-Caballero, Paulino Vico-Rodríguez, Marta Cano-Orihuela, Marcelina Sánchez-Alcalá, María del Carmen Carcelén-Fraile

**Affiliations:** 1 Department of Health Sciences, Faculty of Health Sciences, University of Jaén, 23071 Jaén, Spain; jmmunoz@ujaen.es (J.M.M.-P.);; 2 Department of Health Sciences, Faculty of Health Sciences, University of Atlántico Medio, 35017 Las Palmas de Gran Canaria, Spain; 3 Department of Education Sciences, Faculty of Social Sciences, University of Atlántico Medio, 35017 Las Palmas de Gran Canaria, Spain

**Keywords:** transcranial stimulation, older adults, mild cognitive impairment, systematic review, meta-analysis

## Abstract

**Background/Objectives**: Aging leads to cognitive decline that may progress to dementia. Transcranial direct current stimulation (tDCS) has emerged as a strategy to improve cognitive functions in older adults with mild cognitive impairment (MCI). This study reviews the effectiveness of tDCS in these populations. **Methods**: A systematic review and meta-analysis was conducted following the PRISMA 2020 guidelines. Randomized controlled trials obtained from PubMed, Scopus, Cinahl, and Web of Science were included. Studies with tDCS intervention in older adults with MCI were selected, excluding those without a control group or that did not measure relevant cognitive variables. Methodological quality was analyzed with the PEDro scale and a meta-analysis was applied with random-effects models. **Results**: A total of 27 studies were included in this review, of which 13 were part of the meta-analysis. tDCS showed significant improvements in global cognitive function (*p* < 0.001) and selective attention (*p* = 0.044), but not in mental flexibility or visual attention. Positive effects on quality of life and depressive symptoms were also reported in some studies. **Conclusions**: tDCS may improve cognitive functions in older adults with MCI, but inconsistencies persist in its magnitude and duration. It is recommended to standardize protocols and conduct studies with greater methodological rigor and long-term follow-up.

## 1. Introduction

Population aging represents a growing challenge for health systems and society as a whole [1]. As life expectancy increases, so does the prevalence of age-related cognitive impairments [2], raising the need for effective strategies to preserve brain function and delay cognitive decline [3]. Among the main cognitive problems in the older population are difficulties in memory, processing speed, attention and executive functions, key aspects for the autonomy and quality of life of individuals [4]. Despite advances in the understanding of brain aging, the development of interventions that can mitigate or reverse these effects remains a priority area of research [5].

Within this context, transcranial direct current stimulation (tDCS) has emerged as a promising technique to modulate brain activity and improve cognitive performance in diverse populations [6], including elderly people and patients with mild cognitive impairment (MCI) [7]. tDCS is a noninvasive neuromodulation technique that employs a low-intensity electric current applied through the scalp to alter neuronal excitability in specific areas of the brain [8]. Its mechanism of action is based on the modification of the membrane potential of neurons, facilitating or inhibiting synaptic activity depending on the polarity of the stimulus [9].

The ability of tDCS to modulate cortical excitability has led to its exploration in a wide variety of contexts, including the enhancement of learning, working memory, attention, and executive control [10]. Previous studies have suggested that stimulation applied to regions such as the dorsolateral prefrontal cortex (DLPFC) may generate benefits in working memory and cognitive processing in older adults [11,12]. Furthermore, its application combined with other strategies, such as cognitive training or physical exercise, has shown enhancing effects on brain plasticity [13]. One of the main attractions of tDCS is its safety profile, as it is considered a well-tolerated technique with minimal adverse effects, which are usually limited to mild tingling or itching sensations at the application site [14]. However, its efficacy varies depending on multiple factors, including the intensity and duration of stimulation, the location of the electrode, and individual differences in response to treatment [15]. In addition to its safety and simplicity, an emerging area of interest is the feasibility of tDCS as a home-based therapy. Recent studies suggest that tDCS can be self-administered or applied with the assistance of a caregiver in home settings, with appropriate training and remote supervision protocols [16]. This approach could be especially valuable for older adults with reduced mobility or limited access to specialized care, offering a scalable and cost-effective intervention in cognitive rehabilitation. These findings support the broader applicability of tDCS beyond controlled clinical environments, aligning with the current need for accessible, patient-centered therapeutic options.

Mild cognitive impairment, considered an intermediate stage between normal aging and dementia, has been a key focus of tDCS research [17]. Individuals with MCI have been documented to have alterations in neural networks involved in episodic memory and executive functions, suggesting that modulating these areas through transcranial stimulation may offer significant benefits [18]. Clinical trials have reported improvements in memory tasks and verbal fluency following repeated tDCS sessions, although the magnitude and duration of these effects remain under debate [15,19]. Importantly, emerging evidence highlights that the earlier therapies such as tDCS are administered during the MCI stage, the greater their potential efficacy [20]. Therefore, early identification of suitable candidates becomes crucial. In this context, easily applicable tools such as neuropsychological screening instruments (as reviewed in the present study) and novel blood-based biomarkers—particularly phosphorylated Tau217 and Tau181—may offer valuable support for clinical decision making [21]. While the recent studies already cited [22,23] have indicated that pTau181 and pTau217 in plasma can distinguish amyloid-positive from amyloid-negative individuals, this evidence is significantly strengthened by quantitative syntheses of the literature. Notably, the meta-analysis by van Maurik et al. [24] reported that plasma pTau217 achieves an AUC above 0.90 for identifying amyloid positivity even in cognitively unimpaired individuals. Similarly, the large-scale systematic review by Hansson [25] confirmed the clinical reliability of these biomarkers across all stages of the Alzheimer’s disease spectrum. Additionally, multicenter studies such as Janelidze et al. [26] and Palmqvist et al. [27] found that pTau217 outperforms pTau181 in discriminative accuracy and performs comparably to CSF and PET measures. This body of evidence strongly supports the use of blood-based pTau biomarkers in early detection strategies for identifying patients who may benefit from neuromodulation interventions like tDCS.

Another emerging field in the use of tDCS is its combination with neuropsychological rehabilitation therapies [28]. Recent research has explored the impact of transcranial stimulation on the recovery of cognitive functions in patients with acquired brain damage or neurodegenerative diseases such as Alzheimer’s and Parkinson’s, so these results suggest that tDCS can enhance the effects of cognitive rehabilitation, favoring the consolidation of learning and the reorganization of impaired neural circuits [29]. However, despite the growing interest in tDCS as a tool for cognitive improvement, there are still important challenges in its clinical implementation, since it presents limitations such as the lack of standardized protocols, the heterogeneity in the results of the studies and the need for research with methodologically robust designs that allow evaluating its long-term effectiveness [30].

In view of these considerations, the present review aims to analyze the current evidence on the application of transcranial stimulation in cognitive-related capacities, with special emphasis on its impact on mild cognitive impairment and its potential as an intervention strategy in aging populations.

## 2. Materials and Methods

This review was carried out in accordance with the 2020 PRISMA statement guidelines [31] and the predefined protocol registered in PROSPERO (CRD420250655761). Additionally, the methodological approach followed the recommendations set forth in the “Cochrane Manual for Systematic Reviews of Interventions” [32].

### 2.1. Sources of Information

A literature search was conducted from January to February 2025 using the PubMed, Scopus, Cinahl, and Web of Science (WOS) databases.

### 2.2. Search Strategy

Various keywords were employed in the following search string: (“transcranial direct current stimulation” OR “transcranial current stimulation” OR “tDCS”) AND (“mild cognitive impairment” OR “mild neurocognitive impairment” OR “mci”) AND (“older adults” OR “elderly” OR “aging”).

### 2.3. Inclusion Criteria

The articles chosen had to fulfill these criteria: (i) the studies must be randomized controlled trials (RCTs); (ii) the intervention must involve tDCS as an important part of the treatment; (iii) participants must be from the older population (with an average age of over 60 years); and (iv) participants must have a confirmed diagnosis of MCI using validated diagnostic methods, such as the Montreal Cognitive Assessment (MoCA), the Mini-Mental State Examination (MMSE), Petersen’s criteria, criteria of the MCI Working Group of the European Consortium on Alzheimer’s Disease., diagnosis of aMCI and criteria of the National Institute on Aging-Alzheimer’s Association.

### 2.4. Exclusion Criteria

Articles were excluded if they met any of these criteria: (i) studies without a non-intervention reference group; (ii) studies that did not measure the relevant study variables (cognitive variables such as cognitive status, memory, executive function, and processing speed); (iii) the presence of other conditions such as cancer, stroke, cardiovascular disease, lung disease, and/or kidney disease; and (iv) participants who did not meet the minimum required attendance rate for intervention program sessions (less than two treatment sessions).

### 2.5. Study Selection Process

The study selection process began with the removal of duplicate entries and articles without available abstracts. Titles and abstracts were carefully reviewed to exclude those that did not meet the established eligibility criteria. Articles that passed this initial phase were then evaluated in full text to assess their suitability for inclusion in the meta-analysis. To ensure objectivity and minimize potential bias, two authors (J.C.-S. and J.M.M.-P.) independently carried out the selection process. Any disagreements regarding the eligibility of a study were resolved through consultation with a third author (M.d.C.C.-F.), who provided their judgment to reach a consensus. This thorough procedure ensured that all included studies were relevant and met the predefined criteria.

### 2.6. Data Extraction

The primary variable in this study was the cognitive performance of older patients with mild cognitive impairment. On the other hand, depression and quality of life were also included as secondary variables. Data extraction involved collecting details such as authorship, publication year, study location, population characteristics (sample size, age, and group allocation), study design, outcomes, measurement tools, intervention descriptions, measurement timelines, attrition rates, adverse effects, and key findings.

### 2.7. Assessment of Methodological Quality

Methodological quality was assessed using the PEDro scale [29], which features an 11-item checklist. The maximum score is 10 points, as the first item (“eligibility criteria”) is excluded from the final score. Each item is rated as either “Yes” (1 point) or “No” (0 points). Quality levels are classified as follows: scores between 0 and 3 indicate “Poor” quality, 4 and 5 represent “Fair” quality, 6 to 8 indicate “Good” quality, and scores above 9 are considered “Excellent” [33,34].

The Cochrane RoB-2 tool was also used to assess the risk of bias in each of the selected articles. It is a tool designed to assess the risk of bias in randomized studies, specifically in randomized clinical trials. Its purpose is to assess the risk of bias in randomized studies based on five key domains. The tool classifies the risk of bias of each study as low, high, or unclear [35].

### 2.8. Analytic Decisions for Meta-Analysis

The results are presented in a forest plot, detailing the lead author, year of publication, sample size, individual effects expressed using the Hedge index (g) and the combined effect with its 95% confidence interval, accompanied by the corresponding *p* value. The choice between a fixed-effects or random-effects model will depend on the heterogeneity and variability identified through the Cochrane Q and I^2^ indices. In the meta-analysis, only studies in which the control group received usual care or was assigned to a waiting list were included. For stratified or subgroup analyses, studies were grouped according to the type of intervention implemented, carrying out independent meta-analyses within each category. This strategy facilitated the assessment of variability and effect size in each subgroup, allowing a more detailed interpretation of the findings. Finally, the publication date was analyzed using a funnel plot.

## 3. Results

### 3.1. Study Selection Process

**Figure 1:**
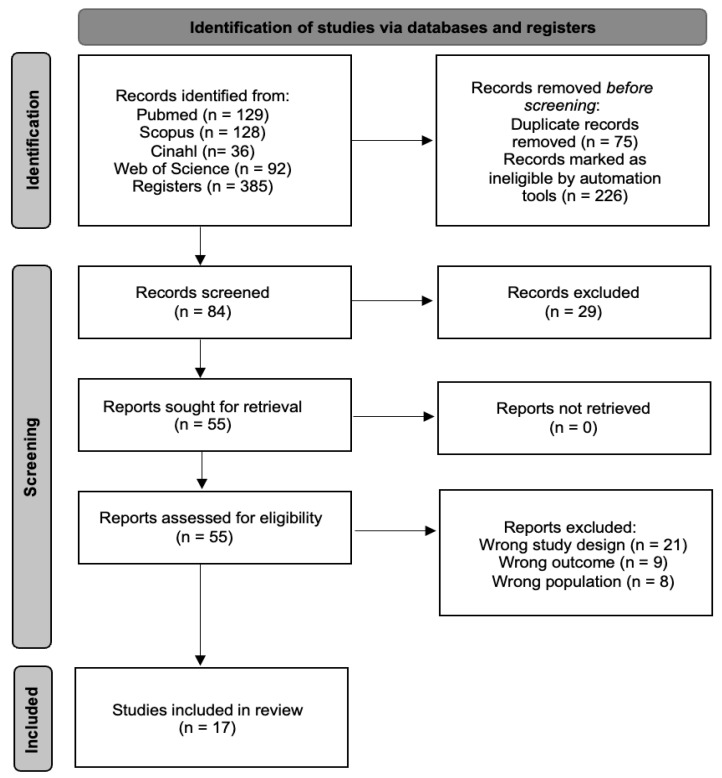
Study selection process flow chart.

An initial search across multiple databases identified 385 articles. The search was then refined within the same databases by targeting specific document types (articles and randomized clinical trials) and filtering for keywords in titles and abstracts, while also removing duplicates. This process resulted in 84 unique articles. These articles were then screened based on their titles and abstracts, narrowing the selection to 55 potential candidates for qualitative evaluation. In the end, 17 articles [36,37,38,39,40,41,42,43,44,45,46,47,48,49,50,51,52] met the inclusion criteria and were included in the meta-analysis, while the remaining 38 were excluded. The selection process is detailed further in Figure 1.

### 3.2. Methodological Quality

The methodological quality of the studies included was assessed using the PEDro scale, with scores obtained from the PEDro website. All twenty-seven were rated as “Good”. To maintain objectivity and consistency in the evaluation, two independent reviewers scored the studies based on the PEDro scale. In cases of disagreement, a conflict resolution procedure was followed. The reviewers discussed the differences, and if they could not reach a consensus, a third reviewer was involved. This approach ensured that study grading was as accurate and reliable as possible. A comprehensive assessment of the methodological quality can be found in Table 1. The risk of bias was also assessed using the Cochrane RoB-2 tool, allowing each article to be scored based on whether the risk of bias was low, unclear, or high. Of the 17 included articles, 5 present a low risk of bias, while the rest are considered to have a low or unclear risk. The full assessment of each section can be seen in Table 2.

### 3.3. Characteristics of the Studies

All the studies included in this systematic review and meta-analysis were randomized controlled trials carried out in Italy [37,44,48], China [38,39,41,43], Germany [36], the United States [42,45,51], South Korea [40,52], Australia [46], Iran [47], Czech Republic [49] and Thailand [50]. A total of 650 participants participated in these studies, with 333 in the control group and 317 in the intervention group, which focused on transcranial stimulation. There is a higher representation of women among the total participants in the studies included in the systematic review. The average age of the participants was 71.5 years (Table 3).

### 3.4. Study Results

Of the 17 articles included in this systematic review, 9 were excluded from the meta-analysis. The article by Lengu et al. [42] was excluded because it focused on evaluating alterations in brain rest and activity, as well as changes in different neurotransmitters. A similar situation occurred with Turnbull et al. [51] and Yun et al. [52], who assessed specific neurological elements observed through imaging tests. Finally, the remaining articles [36,44,46,48,49,50] were excluded for not evaluating global cognitive function, visual attention, mental flexibility, or selective attention as main variables.

The main objective of this systematic review with meta-analysis was to evaluate cognitive abilities. Regarding this evaluation, six articles used the MoCA [38,39,41,42,45,47], while one study employed the MMSE to assess global cognitive function [37]. Four trials used the TMT to study visual attention and mental flexibility. On the other hand, three studies employed the Stroop test to analyze selective attention. Among the studies included in the meta-analysis that assessed global cognition, four reported significant improvements in favor of tDCS [37,39,45,47]. Two of them show results mainly obtained after the treatment, with a tendency to decrease during follow-up, except for the study by Manor et al. [45], in which the results are obtained after the treatment and persist for at least a short follow-up period (two weeks). He et al. [39] only provides data on the variables after treatment. Nevertheless, no article achieves significant results. after treatment or after follow-up for visual attention and mental flexibility, measured using TMT-A and TMT-B, respectively, while two articles [37,40] showed significant changes in selective attention in the post-treatment assessment, achieving a statistical value of *p* < 0.05.

Significant results in cognitive abilities were also observed in the studies excluded from the meta-analysis [44,46,48,50], based on their respective scales measuring specific cognitive aspects and executive function, favoring tDCS. The remaining article [49] did not reach statistical significance (*p* > 0.05) when measuring changes in cognitive abilities after tDCS treatment and after the follow-up. Additionally, neurotransmitter levels and changes observed through neuroimaging were studied, reporting significant effects in favor of tDCS [39,42]. In the following cases [44,46], the results were obtained after treatment and after one or three months of follow-up, respectively, maintaining improvement over time. However, Stonsaovapak et al. evaluate the results only after treatment. Finally, Sandrini et al. [48] reported that the results were observed after treatment and after one month of follow-up, although the positive effects were only sustained in the short term.

In addition, this systematic review also considered secondary variables such as depression and quality of life. Depression was studied in four trials [37,40,46], one of them used the Beck Depression Inventory (BDI) [37], while Kim et al. [40] and Martin et al. [46] employed the Epidemiologic Studies Depression Scale (CESD), Hamilton Rating Scale for Depression (HAMD), and Montgomery Asberg Depression Rating Scale (MADRS). All of them reported significant improvements in favor of tDCS. These results are achieved immediately after treatment [40,47], while Martin et al. [46] obtain them after treatment and during 3 months of follow-up.

Finally, quality of life was analyzed in two studies [46,47] using the Quality of Life Enjoyment and Satisfaction Questionnaire (Q-LES-Q) and the Quality of Life in Alzheimer’s Disease (QoLAD). In this way, Martin et al. [46] did not obtain significant results; however, significant results were achieved by Rezakhani et al. [7]. These results are obtained immediately after treatment and during 3 months of follow-up.

### 3.5. Meta-Analysis

The studies included in this review provided statistical data for four meta-analyses (one for global cognition variables, one for visual attention, one for mental flexibility, and one for selective attention). The findings of each meta-analysis are summarized in Table 4.

#### 3.5.1. Subgroup Analysis

A subgroup analysis was performed considering the study variables. The findings showed a remarkable statistical significance, with moderate and inversely directional Hedge’s g effect sizes. Subgroup analyses based on this assessment tool showed uniform effect sizes in all cases. The consistency of these results indicates that the selection of the assessment tool had a minimal influence on the observed treatment effects.

#### 3.5.2. Global Cognitive Function

**Figure 2:**
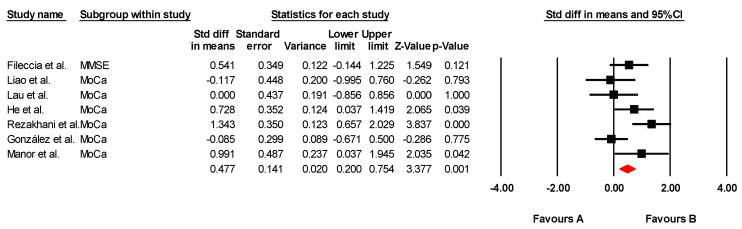
Forest plot of the effectiveness of tDCS on global cognitive function [37,38,39,41,43,45,47].

The effectiveness of tDCS on global cognitive function was assessed across seven studies, including seven independent comparisons, with data from 214 participants. Of these studies, one assessed global cognition using the MMSE and six used the MoCA. Our meta-analysis found low-quality evidence of a large effect in favor of tDCS (SMD = 0.477; 95% CI: 0.200 to 0.754; *p* = 0.001) (Figure 2). There was moderate heterogeneity (I*^2^* = 57.96%; Q = 14.27; df = 6; *p* = 0.027) (Table 5) and no evidence of publication bias (Egger’s test, *p* = 0.88). Sensitivity analyses revealed no variations when individual studies were removed. To assess the clinical relevance of the observed effects, the standardized mean differences (SMDs) were compared with the Minimum Clinically Important Difference (MCID) values reported in the individual studies. In most cases, the improvements in global cognitive function—particularly those measured by MoCA and MMSE—exceeded the MCID thresholds. For instance, studies by De Sousa et al. [36], Fileccia et al. [37], González et al. [38], Lau et al. [41], and Rezakhani et al. [47] reported effect sizes that surpassed the MCID for MoCA (ranging from 0.9 to 1.7 points). This suggests that the observed changes are not only statistically significant, but also clinically meaningful for older adults with MCI.

#### 3.5.3. Visual Attention

**Figure 3:**
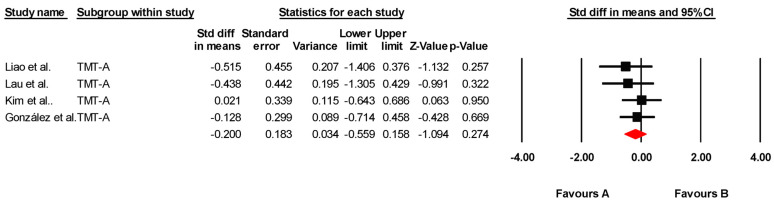
Forest plot of the effectiveness of tDCS on visual attention [38,40,41,43].

The effectiveness of tDCS on visual attention was assessed across four studies, including four independent comparisons, with data from 123 participants. All studies measured visual attention using the Trail Making Test Part A (TMT-A). Our meta-analysis found low-quality evidence of a small, non-significant effect in favor of tDCS (SMD = −0.200; 95% CI: −0.559 to 0.158; *p* = 0.274) (Figure 3). There was no heterogeneity among studies (I*^2^* = 0%; Q = 1.25; df = 3; *p* = 0.740) (Table 6) and no evidence of publication bias (Egger’s test, *p* = 0.15). Sensitivity analyses showed no variations when individual studies were removed. No variations were found by sensitivity analysis.

#### 3.5.4. Mental Flexibility

**Figure 4:**
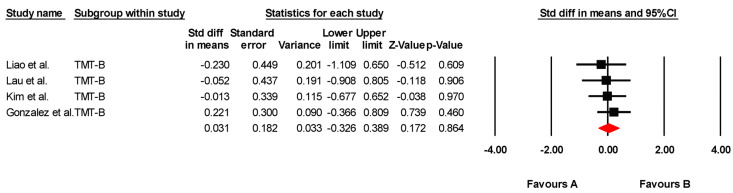
Forest plot of the effectiveness of tDCS on mental flexibility [38,40,41,43].

The effectiveness of transcranial direct current stimulation (tDCS) on mental flexibility was assessed across four studies, including four independent comparisons, with data from 123 participants. All studies measured mental flexibility using the Trail Making Test Part B (TMT-B). Our meta-analysis found low-quality evidence of a very small, non-significant effect in favor of tDCS (SMD = 0.031; 95% CI: –0.326 to 0.389; *p* = 0.864) (Figure 4). There was no heterogeneity among studies (I*^2^* = 0%; Q = 0.79; df = 3; *p* = 0.851) (Table 7) and no evidence of publication bias (Egger’s test, *p* = 0.14). Sensitivity analyses revealed no changes when individual studies were excluded.

#### 3.5.5. Selective Attention

**Figure 5:**
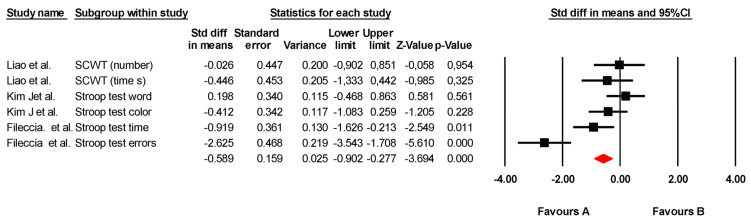
Forest plot of the effectiveness of tDCS on selective attention [37,40,43].

The effectiveness of transcranial direct current stimulation (tDCS) on selective attention was assessed across six independent comparisons from three studies, including data from 305 participants. All studies measured selective attention using variations in the Stroop test. Our meta-analysis found low-quality evidence of a moderate and statistically significant effect in favor of tDCS (SMD = −0.589; 95% CI: −0.902 to −0.277; *p* < 0.001) (Figure 5). There was substantial heterogeneity among the studies (I*^2^* = 81.5%; Q = 27.09; df = 5; *p* = 0.001) (Table 8) but no evidence of publication bias (Egger’s test, *p* = 0.573). Sensitivity analyses revealed no significant variations when individual studies were removed.

## 4. Discussion

The present study aimed to evaluate the efficacy of tDCS in improving cognitive abilities in older adults with MCI. The findings indicate significant improvements in global cognitive function, selective attention, and to a lesser extent, mental flexibility and visual attention. These results support the hypothesis that tDCS can modulate cortical activity and contribute to the preservation of cognitive function in at-risk populations, although discrepancies persist in the magnitude of the effects and their durability.

Global cognitive function refers to the brain’s ability to process, store, and retrieve information necessary for the execution of complex tasks, encompassing multiple cognitive domains such as memory, attention, executive function, language, and visuospatial skills [2]. Its assessment is essential in the early detection of cognitive decline and in the monitoring of interventions aimed at improving brain performance. In this study, global cognitive function was assessed using the MoCA and the MMSE, tests widely used in the detection of mild cognitive impairment and neurodegenerative diseases such as Alzheimer’s disease. Both tests allow estimating general cognitive ability and have been validated in various populations. The results obtained showed significant improvements in MoCA and MMSE scores after the application of tDCS, suggesting a positive effect on cortical activity in key regions such as the dorsolateral prefrontal cortex. Importantly, the clinical relevance of these cognitive improvements was supported by MCID analysis. Several included studies provided sufficient data to estimate whether the observed changes exceeded the minimum threshold to be perceived as beneficial by patients. In the case of MoCA, the majority of studies reported gains exceeding the MCID (typically 1.0–1.5 points), indicating that tDCS led to improvements that were not only statistically detectable but also likely to have a meaningful impact in real-world cognitive functioning. This reinforces the argument for the potential utility of tDCS as a therapeutic tool in clinical settings for older adults with MCI.

This area plays an essential role in regulating working memory, decision making, and cognitive control, so its modulation by tDCS could contribute to the optimization of cognitive performance [7,53] These findings are consistent with previous studies that have reported improvements in general cognitive performance in patients with mild cognitive impairment (MCI) and in healthy older adults after multiple sessions of tDCS [54,55]. In particular, tDCS has been shown to enhance synaptic plasticity, favoring memory consolidation processes and optimizing information processing. A recent meta-analysis also pointed out that tDCS improves working memory and processing speed in older adults, reinforcing the evidence of its therapeutic potential in cognitive aging [56].

Visual attention is the ability to select and process relevant information in dynamic environments, allowing for efficient interaction with the environment. This process involves multiple neural networks, including those related to orientation, alertness, and executive control. In this study, visual attention was assessed using the TMT-A, a widely used test in neuropsychology that measures visual processing speed and sustained attention [3]. This test consists of connecting a series of numbers in sequential order as quickly as possible, allowing for the assessment of cognitive efficiency in tasks that require visual exploration and visuomotor coordination. The results of this study indicated improvements in execution times after the application of tDCS, suggesting a possible beneficial effect on processing speed and attentional efficiency. However, these differences did not reach statistical significance, which could be due to interindividual variability in response to stimulation or the need for longer or more intensive protocols. Although previous research has suggested that stimulation of the parietal cortex can influence attentional networks, the current evidence remains inconclusive [8]. Some studies have shown that tDCS can induce changes in functional connectivity between regions involved in perception and decision making, facilitating the selection of relevant information in complex visual tasks. These findings are relevant given that aging entails a decline in attentional functions, which can affect autonomy and performance in daily activities. However, other studies have found that the effects of tDCS may be short-lived if repeated sessions are not conducted to reinforce changes in cortical activity and consolidate long-term benefits. Therefore, future studies should explore longer stimulation protocols or combinations with other interventions to optimize the effects on visual attention and processing speed [57,58].

Mental flexibility is the ability to switch between different tasks or cognitive rules efficiently, allowing individuals to adapt to new situations, solve problems effectively, and modify strategies according to environmental demands [59]. This ability is a key component of executive functions and is closely related to decision making, planning, and cognitive control. Its assessment was performed using the TMT-B, a widely used neuropsychological test that measures the ability to switch between different stimulus sets, thereby assessing processing speed, working memory, and divided attention. The results of this study indicated only a minimal effect in participants who received tDCS, and this was not statistically significant. Variability across studies suggests that this effect is not uniform and may depend on multiple factors, such as electrode location, current intensity, and individual differences in brain plasticity. Previous studies have found that stimulation of the DLPFC can improve cognitive flexibility by increasing functional connectivity in executive networks, favoring the regulation of attention and the inhibition of automatic responses [60]. However, other studies have not replicated these findings, suggesting that mental flexibility may be less sensitive to tDCS stimulation than other cognitive functions, such as working memory or response inhibition [61]. This has led researchers to explore possible moderating variables, such as the duration of the intervention or the combination with other cognitive training strategies. Recent research has indicated that combining tDCS with structured cognitive training programs can significantly enhance the benefits in mental flexibility, maximizing the transfer of improvements to daily life and performance in complex tasks [62]. These findings reinforce the need for multimodal approaches, integrating brain stimulation with behavioral interventions and personalized strategies to optimize their effects on cognition.

Selective attention refers to the ability to focus on relevant information while inhibiting interference from distractors, playing a key role in executive control [10]. This ability is essential for efficient information processing in environments with multiple stimuli, and its alteration can significantly affect cognitive performance and decision making. In this study, it was assessed using the Stroop test, a task widely used in cognitive neuroscience to measure the ability to inhibit automatic and impulsive responses. Significant improvements in response times and accuracy were observed after the application of tDCS, suggesting a positive effect in the modulation of neural circuits associated with cognitive control. These findings are consistent with recent studies reporting that tDCS applied to the prefrontal cortex improves performance in Stroop tasks. However, the size of the effect varies according to factors such as age, cognitive status of the participant, and specific stimulation parameters [63]. Some studies suggest that tDCS may be particularly beneficial in people with more advanced cognitive impairment, possibly by facilitating synaptic plasticity and functional connectivity in affected neural networks [64]. Likewise, research has indicated that stimulation can influence the release of neurotransmitters such as dopamine, contributing to better regulation of executive control and cognitive flexibility. However, some studies have not found significant differences between stimulated and control groups, highlighting the need for clinical trials with more rigorous methodological designs, considering variables such as stimulation duration, current intensity, and individual differences in response to tDCS [56]. These findings underline the importance of continuing to explore the underlying mechanisms of tDCS and its potential application in clinical and neuroscientific settings.

Despite the positive findings, this study has several limitations. The heterogeneity in stimulation protocols makes it difficult to directly compare results across studies, highlighting the need to standardize tDCS application parameters. Furthermore, most of the studies included in this review have relatively small sample sizes, limiting the generalizability of the results. The durability of the effects also remains uncertain, as few trials have assessed the long-term impact of tDCS on cognitive function. Furthermore, due to the limited number of studies included in some subgroup analyses, funnel plots were not generated when fewer than 10 studies were available, in accordance with standard recommendations. Even when 11 studies are included, the funnel plot’s ability to detect publication bias has been shown to be limited, so the results should be interpreted with caution. It is also worth noting that mild cognitive impairment (MCI) does not inevitably progress to dementia in all individuals. Some cases may remain stable or even improve spontaneously over time. Therefore, while the improvements in cognitive function observed in this review are encouraging, they should be considered surrogate indicators of potential clinical benefit. Further research is needed to determine whether these effects translate into reduced rates of progression to dementia in the long term. Finally, although tDCS is a safe and well-tolerated technique, further studies are required to explore its effect in different patient subgroups and to analyze possible interactions with other cognitive intervention strategies. Clinical trials with more robust methodologies and long-term follow-ups are recommended to more accurately assess the efficacy and safety of tDCS in the treatment of mild cognitive impairment.

## 5. Conclusions

The results of this review and meta-analysis indicate that tDCS can improve global cognitive function and selective attention in older adults with MCI. These findings suggest that tDCS has the potential to modulate brain activity and promote cognitive performance in at-risk populations. Although the efficacy of tDCS varies depending on the cognitive function assessed, the results support its use as a potential therapeutic tool. Standardization of application protocols and combination with other intervention strategies could optimize its benefits. Therefore, tDCS is presented as a promising alternative for the treatment of cognitive impairment, with the need to continue researching its implementation and long-term effects.

## Figures and Tables

**Table 1:** Methodological Quality of the included articles.

	1	2	3	4	5	6	7	8	9	10	11	Total Score
De Sousa et al. [36]	1	1	0	1	1	0	0	1	0	1	1	6
Fileccia et al. [37]	1	1	0	1	0	0	1	1	1	1	1	8
González et al. [38]	1	1	1	1	1	0	1	1	1	1	1	9
He et al. [39]	1	1	1	1	1	0	1	1	1	1	1	9
Kim et al. [40]	1	1	1	1	1	0	1	1	1	1	1	9
Lau et al. [41]	1	1	1	1	1	0	1	1	1	1	1	9
Lengu et al. [42]	1	1	1	1	1	0	1	1	1	1	1	9
Liao et al. [43]	1	1	1	1	1	0	1	1	1	1	1	9
Manenti et al. [44]	1	1	1	1	1	0	1	1	1	1	1	9
Manor et al. [45]	1	1	0	1	1	1	0	1	0	1	1	7
Martin et al. [46]	1	1	1	1	1	0	1	1	1	1	1	9
Rezakhani et al. [47]	1	1	1	1	1	0	1	1	1	1	1	9
Sandrini et al. [48]	1	1	0	1	1	0	1	1	1	1	1	8
Šimko et al. [49]	1	1	1	1	1	1	0	1	1	1	0	8
Stonsaovapak et al. [50]	1	1	1	1	1	0	1	1	1	1	1	9
Turnbull et al. [51]	1	1	0	1	1	0	1	1	1	1	1	8
Yun et al. [52]	1	1	1	1	1	0	1	1	1	1	0	8
Sum of column scores.	17	17	12	17	16	2	14	17	15	17	15	-

Items: 1: eligibility criteria; 2: random allocation; 3: concealed allocation; 4: baseline comparability; 5: blind subjects; 6: blind therapists; 7: blind assessors; 8: adequate follow-up; 9: intention-to-treat analysis; 10: between-group comparisons; 11: point estimates and variability; yes = 1; no = 0.

**Table 2:** RoB-2 to assess the risk of bias.

Bias	Bias in Randomization	Bias Due to Deviations from the Intervention.	Bias Due to Missing Data	Bias in the Measurement of Outcomes	Bias in the Selection of Reports	Overall Assessment of the Risk Bias
De Sousa et al. [36]	Low risk of bias	Low risk of bias	Unclear risk of bias	Unclear risk of bias	Low risk of bias	Low or unclear
Fileccia et al. [37]	Low risk of bias	Low risk of bias	Unclear risk of bias	Unclear risk of bias	Low risk of bias	Low or unclear
González et al. [38]	Low risk of bias	Low risk of bias	Unclear risk of bias	Low risk of bias	Low risk of bias	Low or unclear
He et al. [39]	Low risk of bias	Low risk of bias	Low risk of bias	Low risk of bias	Low risk of bias	Low
Kim et al. [40]	Low risk of bias	Unclear risk of bias	Unclear risk of bias	Low risk of bias	Low risk of bias	Low or unclear
Lau et al. [41]	Low risk of bias	Unclear risk of bias	Unclear risk of bias	Low risk of bias	Low risk of bias	Low or unclear
Lengu et al. [42]	Low risk of bias	Unclear risk of bias	Unclear risk of bias	Low risk of bias	Low risk of bias	Low or unclear
Liao et al. [43]	Low risk of bias	Unclear risk of bias	Unclear risk of bias	Low risk of bias	Low risk of bias	Low or unclear
Manenti et al. [44]	Low risk of bias	Unclear risk of bias	Unclear risk of bias	Low risk of bias	Low risk of bias	Low or unclear
Manor et al. [45]	Low risk of bias	Unclear risk of bias	Low risk of bias	Low risk of bias	Low risk of bias	Low or unclear
Martin et al. [46]	Low risk of bias	Low risk of bias	Low risk of bias	Low risk of bias	Low risk of bias	Low
Rezakhani et al. [47]	Low risk of bias	Low risk of bias	Low risk of bias	Low risk of bias	Low risk of bias	Low
Sandrini et al. [48]	Low risk of bias	Low risk of bias	Low risk of bias	Low risk of bias	Low risk of bias	Low
Šimko et al. [49]	Low risk of bias	Unclear risk of bias	Unclear risk of bias	Low risk of bias	Low risk of bias	Low or unclear
Stonsaovapak et al. [50]	Low risk of bias	Low risk of bias	Low risk of bias	Low risk of bias	Low risk of bias	Low
Turnbull et al. [51]	Low risk of bias	Low risk of bias	Unclear risk of bias	Low risk of bias	Low risk of bias	Low or unclear
Yun et al. [52]	Unclear risk of bias	Low risk of bias	Low risk of bias	Low risk of bias	Low risk of bias	Low or unclear

**Table 3:** Characteristics of the included studies.

Author	Study Design	Sample CG/IG and Sex	Control Group	Age CG/IG	Treatment	Adverse Events or Side Effects	Placement of the Electrodes	Parameters Intervention Group	Results
De Sousa et al. [36]	RCT Single-blind cross-over, placebo controlled	24/24 F: 56.25% M: 43.75%	visuospatial OLM training plus sham atDCS	69 (7)/70 (6)	visuospatial OLM training plus atDCS	No adverse events	Anode: right temporoparietal cortex Catode: left supraorbital region	F: 3 times/week #S: 3 sessions D: 20 min CI: 1 mA	OLM training combined with atDCS improved training success only in MCI patients. The relative performance improvement was similar between MCI patients and HE participants with atDCS. No positive effect was observed after one month. Exploratory analyses indicated a positive effect on online performance but a negative effect on offline performance in MCI patients. In both groups, post hoc exploratory analyses revealed that individuals with initially low performance showed a greater benefit from atDCS.
Fileccia et al. [37]	RCT, single-blind, parallel, placebo-controlled	17/17 F: 29.4% M: 70.6%	Sham tDCS	69.7 (1.6)/71.6 (1.4)	tDCS	Slight itching sensation	Anode: left DLPFC Catode: right deltoid muscle	F: 5 times/week #S: 20 sessions D: 20 min CI: 2 mA	At follow-up, patients who received anodal stimulation demonstrated improvements in episodic verbal memory (*p* < 0.001), figure naming (*p* < 0.01), overall cognitive function (Brief Mental Deterioration Battery) (*p* < 0.0001), and mood (Beck Depression Inventory) (*p* < 0.01).
González et al. [38]	RCT, double-blind, parallel, placebo-controlled	45/22 F: 72.7% M: 27.3%	Sham tDCS combined with CT	71 (6.2)/69.8 (5.3)	tDCS combined with CT	Discomfort in two patients	Anode: left DLPFC Catode: contraleral braquioradialis muscle	F: 3 time/week #S: 9 sessions D: 30 min CI: 1.5 mA	All three groups showed improvements in global cognition and everyday memory (*p* < 0.017) both after the intervention and at follow-up, with the tDCS + CT group showing larger effect sizes (d > 0.94) compared to the other groups, though no significant differences were found between the groups. Regarding CT outcomes, significant differences were observed in favor of the tDCS + CT group, particularly in reducing the completion and reaction times for working memory and attention tasks (*p* < 0.017).
He et al. [39]	RCT, double-blind, parallel, placebo-controlled	19/24 F: 74.4% M: 25.6%	Sham tDCS	65.63 (3.53)/63.5 (4.8)	HD-tDCS	Headache, scalp pain, tingling, itching, burning, skin redness, difficulty concentrating, skin lesions, and drowsiness	Anode: left DLPFC Cathode: surrounding the anode in a 4 × 1 montage	F: 5 times/week #S: 10 sessions D: 20 min CI: 1 mA	The findings revealed that the fALFF and ReHo values were altered in various brain areas following HD-tDCS. Significant decreases in fALFF values were observed in the right Insula, right Precuneus, left Thalamus, and right Superior Parietal regions, while the right Inferior Temporal, left Fusiform, left Superior Occipital, right Calcarine, and right Angular regions exhibited notable increases in fALFF values. Significant increases in ReHo values were found in the right Inferior Temporal, left Putamen, left Middle Frontal, right Precentral, left Medial Superior Frontal, right Superior Frontal, and left Precentral regions. These results suggest that HD-tDCS can modify both the intensity and synchronization of brain activity, and that fALFF and ReHo analyses are effective tools for detecting changes in spontaneous brain activity following HD-tDCS.
Kim et al. [40]	RCT, double-blind, parallel, placebo-controlled	23/14 F: 64.9% M: 35.1%	Sham stimulation	73.1 (6.3)/76.1 (7.4)	Home-based and remotely monitored tDCS	Discomfort in some patients	Anode: left DLPFC Cathode: right DLPFC	F: 5 times/week #S: 30 sessions D: 30 min CI: 2 mA	In terms of the effects on both depressive symptoms and cognitive functions, active tDCS did not show a significant difference compared to sham tDCS. However, when compared to sham stimulation, active tDCS resulted in a decrease in delta frequency activation and an increase in beta frequency activation. Additionally, the increase in beta activity was linked to cognitive improvement, but only in the active group.
Lau et al. [41]	RCT, double-blind, parallel, placebo-controlled	10/11 F: 66.7% M: 33.3%	Sham tDCS and ICCT	69 (4.9)/72 (17.3)	tDCS and ICCT	No adverse events	Anode: left DLPFC Cathode: right supraorbital region	F: 3 times/week #S: 15 sessions D: 20 min, 40 min with cognitive training CI: 2 mA	Both groups showed improvements in global cognition, executive function, and working memory scores, but no significant interaction effects were found on cognitive outcomes. Furthermore, the group × time interactions revealed that tDCS + ICCT significantly improved dual-task gait performance, specifically in gait speed (*p* = 0.045), variability (*p* = 0.016), and dual-task cost (*p* = 0.039), when compared to sham + ICCT.
Lengu et al. [42]	RCT, double-blind, cross-over, placebo-controlled	19/13 F: 37.5% M: 62.5%	Sham HD-tDCS	69.26 (6.73)/71.15 (5.26)	Active HD-tDCS	No adverse events	Anode: RSPC Cathode: 4 × 1 montage	F: 2 times/week #S: 2 sessions D: 20 min CI: 2 mA	Compared to the sham condition, and after adjusting for MRS voxel overlap and right superior parietal volume, active HD-tDCS significantly increased GABA levels and decreased the glutamate-to-GABA ratio. No changes were noted in the left prefrontal control MRS voxel. While no significant correlation was found between the strength of the delivered current (measured through MRI-based computational modeling) and changes in neurometabolites, a strong positive relationship was observed between the volume of the right superior parietal cortex and changes in neurometabolites.
Liao et al. [43]	RCT, double-blind, parallel, placebo-controlled	10/10 F: 65% M: 35%	Sham tDCS and Tai Chi	73.1 (4.6)/72.6 (4.1)	Anodal tDCS and Tai Chi	No adverse events	Anode: left DLPFC Cathode: right supraorbital region	F: 3 times/week #S: 36 sessions D: 20 min, 40 min with Tai Chi CI: 2 mA	Significant interaction effects between groups were observed in the cognitive dual-task walking. The anodal tDCS group showed a greater improvement in cadence and dual-task cost of speed compared to the sham group. Combining tDCS with Tai Chi may provide additional benefits over TC alone in improving dual-task gait performance in patients with MCI.
Manenti et al. [44]	RCT, double-blind, parallel, placebo-controlled	9/9 F: 45.5% M: 55.5%	Sham tDCS	75.3 (2.2)/75.3 (4.8)	Active tDCS	Itching and irritation with light to moderate intensity	Anode: left LPFC Cathode: right supraorbital region	F: 3 times/week #S: 3 sessions D: 15 min CI: 1.5 mA	There were no significant differences in word recall performance between the active and sham groups after the last learning trial on Day 1 (*p* = 0.17), indicating similar baseline performance. However, the recognition task analysis showed a significant “Group” effect (*p* = 0.044), with the active tDCS group performing better than the sham group, and a significant “Time” effect (*p* = 0.020), showing decreased performance from Day 3 to Day 30. The interaction between “Group” and “Time” was not significant (*p* = 0.922). No significant effects were found for the “Group” (*p* = 0.563), “Time” (*p* = 0.826), or their interaction (*p* = 0.293) on the C criterion of the recognition task. Finally, the word recall analysis showed a significant “Time” effect (*p* = 0.041), indicating performance decline from Day 3 to Day 30, while “Group” (*p* = 0.626) and the interaction between “Group” and “Time” (*p* = 0.553) were not significant.
Manor et al. [45]	RCT, double-blind, parallel, placebo-controlled	10/9 F: 52.6% M: 47.4%	Sham tDCS	79 (4)/82 (4)	tDCS	Sensations under the electrode, skin redness, drowsiness and headache	Anode: left DLPFC (Brodmann Area 46) Cathode: right supraorbital region	F: 5 times/week #S: 10 sessions D: 20 min CI: 2 mA	Compared to sham, tDCS led to improvements in the total MoCA score (*p* = 0.03), particularly in the executive function sub-score (*p* = 0.002), as well as in several dual-task standing and walking metrics (*p* < 0.05). These effects lasted for two weeks. tDCS had no impact on the TUG for mobility or the Geriatric Depression Scale. Participants who showed greater improvements in dual-task standing posture after the first tDCS session also demonstrated larger cognitive-motor improvements after two weeks of tDCS (*p* < 0.04).
Martin et al. [46]	RCT, double-blind, parallel, placebo-controlled	35/33 F: 66.2% M: 33.8%	CT + sham tDCS	71.6 (6.35)/71.8 (6.39)	CT + active tDCS	Tingling, redness, mild burning and itching	Anode: left DLPFC Catode: right VLPFC	F: 3 times/week #S: 15 sessions D: 30 min CI: 2 mA	The CT + active tDCS group showed significant improvement post-treatment (*p* = 0.033), whereas the CT + sham tDCS group did not (*p* = 0.050), although there was no difference between the groups. At the 3-month follow-up, both groups exhibited significant memory improvements compared to pre-treatment (CT + active tDCS: *p* < 0.01; CT + sham tDCS: *p* < 0.01), but no significant difference was found between the groups.
Rezakhani et al. [47]	RCT, double-blind, parallel, placebo-controlled	20/40 F: 36.7% M: 63.3%	Sham HD-tDCS	69.35 (9.94)/68.65 (10.09)	Left DLPFC or DATL HD-tDCS	No adverse events	Anode group 1: left DLPFC Anode group 2: DATL Cathode both groups: right prefrontal region	F: 5 times/week #S: 10 sessions D: 20 min CI: 2 mA	MCI patients showed the highest MoCA mean scores in both the left DLPFC and DATL groups compared to the study baseline, 2 weeks after the intervention. Additionally, the MoCA mean scores for MCI patients were higher in both intervention groups than in the sham group up to 3 months post-stimulation (*p* ≤ 0.05). However, a decreasing trend in MoCA mean scores was observed as time progressed from the initial stimulation. Furthermore, higher QoLAD mean scores were seen in the left DLPFC and DATL groups 3 months post-stimulation, emphasizing the effectiveness of anodal HD-tDCS in enhancing the quality of life in MCI patients.
Sandrini et al. [48]	RCT, double-blind, parallel, placebo-controlled	14/14 F: 60.7% M: 39.3%	Sham tDCS	69.1 (3.4)/68.6 (4.2)	Anodal tDCS	Itching and irritation with light to moderate intensity	Anode: left LPFC Cathode: right supraorbital region	F: 3 times/week #S: 3 sessions D: 15 min CI: 1.5 mA	The results demonstrated that anodal tDCS enhanced episodic memory, as evidenced by improved delayed recall (48 h) compared to placebo stimulation. The finding that PFC-tDCS during learning can improve verbal episodic memory in the elderly suggests the potential for designing targeted neurorehabilitation protocols for conditions that impact episodic memory, such as mild cognitive impairment.
Šimko et al. [49]	RCT, double-blind, parallel, placebo-controlled	18/17 F: - M: -	Sham stimulation	72.4 (4.96) both groups	tDCS-cog	No adverse events	Anode: left DLPFC Cathode: left Middle Frontal gyrus	F: 5 times/week #S: 10 sessions D: 20 min CI: 2 mA	Our main finding showed that tDCS-cog did not produce superior after-effects compared to the sham on VOMT in individuals with MCI, as indicated by insignificant immediate and long-lasting effects. Moreover, tDCS-cog did not enhance honline training as expected. The fMRI analysis revealed changes in brain activity in the left insula, which may be associated with the tDCS-cog intervention.
Stonsaovapak et al. [50]	RCT, double-blind, parallel, placebo-controlled	22/23 F: 91.1% M: 8.9%	Placebo stimulation	69.68 (7.6)/68.39 (8.37)	atDCS	Tingling and itching sensation	Anode: left DLPFC Cathode: right supraorbital region	F: 3 times/week #S: 12 sessions D: 20 min CI: 2 mA	CANTAB results showed a significant improvement in VSA accuracy in the atDCS group at all three time points, as well as improvements in SWM and VM immediately after the first stimulation, along with a reduced VM reaction time after 12 sessions. Long-lasting effects on VSA and VM were observed 4 weeks post-treatment.
Turnbull et al. [51]	RCT, double-blind, parallel, placebo-controlled	20/20 F: 60% M: 40%	Sham tDCS	73 (7.1)/70 (6.6)	Anodal tDCS	Tingling and itching	Anode: left SMC Cathode: right orbitofrontal region	F: 5 times/week #S: 14 sessions D: 20 min CI: 1.5 mA	Generalized Estimating Equations showed no significant group-by-time interactions for either NPS measure. However, there was evidence of a reduction in patient-reported NPS (*p* = 0.051), decreased LSMC activation during visual attention (*p* = 0.087), and increased LSMC-amygdala resting-state functional connectivity (*p* = 0.077) in the intervention group from pre- to post-intervention. The decrease in LSMC activation (*p* = 0.002) and the increase in LSMC-amygdala rsFC (*p* = 0.030) were associated with the reduction in patient-reported NPS. Additionally, increased positive valence across sessions was significantly linked to NPS improvement related to the intervention (*p* < 0.001). No findings were observed for caregiver-reported NPS. The effects were more pronounced in the left postcentral gyrus compared to the left Precentral gyrus.
Yun et al. [52]	RCT, double-blind, parallel, placebo-controlled	18/17 F: 68.7% M: 31.3%	Sham tDCS	73.12 (4.25)/74.75 (7.47)	tDCS	No adverse events	Anode: left DLPFC Cathode: right DLPFC	F: 3 times/week #S: 9 sessions D: 30 min CI: 2 mA	We demonstrated that consistent and prolonged use of tDCS significantly boosted regional cerebral metabolism in MCI patients. Additionally, improvements in subjective memory satisfaction and memory strategies were only seen in the real tDCS group after 3 weeks of stimulation.

RCT: randomized controlled trial; CG: control group; IG: intervention group; CT: cognitive training; OLM: associative Object-Location Memory; MCI: Mild Cognitive Impairment; atDCS: anodal transcranial direct current stimulation; tDCS: transcranial direct current stimulation; HD-tDCS: high-definition transcranial direct current stimulation; fALFF: fractional Amplitude of Low-Frequency Fluctuation; ReHo: Regional Homogeneity; CCT: Cognitive Control Training; ICCT: Interactive Computerized Cognitive Training; MRS: Magnetic Resonance Spectroscopy; GABA: Glutamate and Gamma-aminobutyric Acid; MoCa: Montreal Cognitive Assessment; TUG: Timed Up-and-Go test; MMSE: Mini Mental State Exam; TMT-A: Trail Making Test-A; DLPFC: dorsolateral prefrontal cortex; DATL: Dominal Anterior Temporal Lobe; PFC-tDCS: prefrontal cortex transcranial direct current stimulation; RSPC: right Superior Parietal Cortex; LPFC: lateral prefrontal cortex; VLPFC: ventrolateral prefrontal cortex; SMC: Sensorimotor Cortex; tDCS-cog: transcranial direct current stimulation combined with cognitive training; VOMT: Visual Object Matching Task; CANTAB: Cambridge Neuropsychological Test Automated Battery; VSA: Visual Sustained Attention; SWM: Spatial Working Memory; VM: Visual Memory; rsFC: resting-state functional connectivity; NPS: Neuropsychiatric Symptoms; F: frequency; #S: number of sessions; D: duration; CI: Current Intensity; mA: milliamperes.

**Table 4:** Main findings in meta-analyses.

Effect Size							Heterogeneity		Publication Bias		
Variables	K	N	N_s_	SMD	95% CI	*p*	Q (df)	I^2^ (*p*)	Funnel Plot (Egger *p*)	Trim and Fill	
										Adj SMD	% var
Global cognitive function	7	214	30.6	0.477	0.200 to 0.754	0.001	6	57.96	0.88	0.48	14.27%
Visual attention	4	123	30.8	−0.200	−0.559 to 0.158	0.274	3	0.000	0.15	−0.20	1.25%
Mental flexibility	4	123	30.8	0.031	−0.326 to 0.389	0.864	7	0.000	0.11	0.03	0.79%
Selective attention	3	91	30.3	0.682	−1.418 to 0.054	0.069	5	81.540	0.32	−0.81	37.84%

Abbreviations: K, number of comparisons; N, total sample size; Ns, mean number of participants per study; SMD, standardized mean difference; 95% CI. 95% confidence interval; *p*, *p*-value; Q, Q-test; df, degree of freedom; I^2^, degree of inconsistency.

**Table 5:** Study characteristics and heterogeneity statistics for global cognitive function.

Study Name	Subgroup Within Study	Experimental Group			Control Group			Heterogeneity			
		*n*	M	SD	n	M	SD	Q-Value	df (Q)	*p*-Value	I^2^
Fileccia et al. [37]	MMSE	17	26.6	0.50	17	17.0	25.1	14.27	6	0.0027	57.96
Liao et al. [43]	MoCa	10	24.9	3.62	10	25.3	3.19				
Lau et al. [41]	MoCa	11	26.6	17.0	10	26.7	2.20				
He et al. [39]	MoCa	20	26.7	4.10	15	24.0	3.10				
Rezakhani et al. [47]	MoCa	20	27.0	2.55	20	23.7	2.36				
González et al. [38]	MoCa	21	26.2	2.00	24	26.4	2.60				
Manor et al. [45]	MoCa	9	25.0	4.00	10	22.0	1.76				

**Table 6:** Study characteristics and heterogeneity statistics for visual attention.

Study Name	Subgroup Within Study	Experimental Group			Control Group			Heterogeneity			
		n	M	SD	n	M	SD	Q-Value	df (Q)	*p*-Value	I^2^
Liao et al. [43]	TMT-A	10	44.8	8.99	10	51.8	17.0	1.25	3	0.740	0.000
Lau et al. [41]	TMT-A	11	48.3	8.90	10	52.5	10.3				
Kim et al. [40]	TMT-A	14	42.3	25.8	23	41.8	22.1				
González et al. [38]	TMT-A	21	45.5	18.1	24	48.4	26.0				

**Table 7:** Study characteristics and heterogeneity statistics for mental flexibility.

Study Name	Subgroup Within Study	Experimental Group			Control Group			Heterogeneity			
		n	M	SD	n	M	SD	Q-Value	df (Q)	*p*-Value	I^2^
Liao et al. [43]	TMT-B	10	88.3	35.6	10	95.9	30.4	0.79	3	0.851	0.000
Lau et al. [41]	TMT-B	11	91.4	32.5	10	92.8	19.4				
Kim et al. [40]	TMT-B	14	86.0	34.7	23	86.4	28.8				
González et al. [38]	TMT-B	21	72.5	34.1	24	66.0	24.5				

**Table 8:** Study characteristics and heterogeneity statistics for selective attention.

Study Name	Subgroup Within Study	Experimental Group			Control Group			Heterogeneity			
		n	M	SD	n	M	SD	Q-Value	df (Q)	*p*-Value	I^2^
Liao et al. [43]	SCWT (number)	10	32.2	5.34	10	32.4	9.53	27.09	5	0.001	81.5
Liao et al. [43]	SCWT (time’s)	10	67.3	11.70	10	74.2	18.50				
Kim et al. [40]	Stroop test word	14	96.5	25.80	23	91.1	28.20				
Kim et al. [40]	Stroop test color	14	52.1	26.70	23	63.4	27.80				
Fileccia et al. [37]	Stroop test time	17	22.1	4.50	17	28.7	9.10				
Fileccia et al. [37]	Stroop test errors	17	0.3	0.70	17	6.2	3.10				

## Data Availability

Not applicable.

## Abbreviations

The following abbreviations are used in this manuscript:

tDCS	transcranial direct current stimulation
MCI	mild cognitive impairment
WOS	Web of Science
RCTs	randomized controlled trials
MoCA	Montreal Cognitive Assessment
MMSE	Mini-Mental State Examination
CT	cognitive training
OLM	associative Object-Location Memory
atDCS	anodal transcranial direct current stimulation
HD-tDCS	high-definition transcranial direct current stimulation
fALFF	fractional Amplitude of Low-Frequency Fluctuation
ReHo	Regional Homogenity
CCT	Cognitive Control Training
ICCT	Interactive Computerized Cognitive Training
MRS	Magnetic Resonance Spectroscopy
GABA	Glutamate and Gamma-aminobutyric Acid
TUG	Timed Up-and-Go test
TMT-A	Trail Making Test-A
DLPFC	dorsolateral prefrontal cortex
DATL	Dominal Anterior Temporal Lobe
PFC-tDCS	prefrontal cortex transcranial direct current stimulation
tDCS-cog	transcranial direct current stimulation combined with cognitive training
VOMT	Visual Object Matching Task
CANTAB	Cambridge Neuropsychological Test Automated Battery
VSA	Visual Sustained Attention
SWM	Spatial Working Memory
VM	Visual Memory
SCWT	Stroop Color Word Test
rsFCNPS	functional connectivity Neuropsychiatric Symptoms
F	frequency
#S	number of sessions
D	duration
